# What is new in the classification of peripheral T cell lymphomas?

**DOI:** 10.1007/s00292-023-01260-y

**Published:** 2023-12-04

**Authors:** Laurence de Leval, Bettina Bisig

**Affiliations:** Institute of Pathology, Department of Laboratory Medicine and Pathology, Lausanne University Hospital (CHUV) and Lausanne University, 25 rue du Bugnon, 1011 Lausanne, Switzerland

**Keywords:** Follicular helper T cell lymphoma, Anaplastic large cell lymphomas, Intestinal T and NK cell lymphomas, Mutations, Cell of origin, Follikuläres T-Helfer-Zell-Lymphom, Anaplastische großzellige Lymphome, Intestinale T- und NK-Zell-Lymphome, Mutationen, Ursprungszelle

## Abstract

In this review focus article, we highlight the main modifications introduced in the latest 2022 International Consensus Classification and World Health Organization classification (ICC and WHO-HAEM5) of mature T (and NK) cell neoplasms (PTCLs) and consequent implications for diagnostic practice. The changes result from recent advances in the genomic and molecular characterization of PTCLs and enhanced understanding of their pathobiology. Specifically, consideration is given to the following groups of diseases: Epstein–Barr virus (EBV)-associated neoplasms; follicular helper T cell lymphoma; anaplastic large cell lymphomas; primary intestinal T and NK cell lymphomas and lymphoproliferative disorders; and PTCL, not otherwise specified.

## Abstract (de)

In dem vorliegenden Übersichtsbeitrag liegt der Schwerpunkt auf den wesentlichen Modifikationen der aktuellen, 2022 eingeführten Klassifikation reifer T‑ (und NK-)Zell-Neoplasien, also peripherer T‑Zell-Lymphome (PTCL) gemäß International Consensus Classification (ICC) und der Weltgesundheitsorganisation-Klassifikation (5. Auflage, WHO-HAEM5) sowie auf den daraus folgenden Auswirkungen auf die praktische Diagnostik. Die Veränderungen beruhen auf aktuellen Fortschritten in der genomischen und molekularen Charakterisierung der PTCL und einem vertieften Verständnis ihrer Pathobiologie. Insbesondere werden die folgenden Krankheitsgruppen berücksichtigt: Epstein-Barr-Virus(EBV)-assoziierte Neoplasien, follikuläres T‑Helfer-Zell-Lymphom, anaplastische großzellige Lymphome, primäre intestinale T- und NK-Zell-Lymphome sowie lymphoproliferative Erkrankungen, außerdem nicht anderweitig spezifizierte PTCL.

Neoplasms derived from mature NK or T cells (peripheral T cell lymphomas, PTCLs) are rare overall, but encompass diverse clinical presentations of diseases ranging from uncommonly indolent to usually aggressive. The two classifications of lymphoid neoplasms developed in 2022, namely the International Consensus Classification (ICC) [5] and the fifth edition of the World Health Organization (WHO) classification (WHO-HAEM5) [1], represent updates of the 2017 revised fourth WHO classification (WHO-HAEM4R) (Fig. 1), and rely on a multiparametric definition of lymphoma entities. Recent advances in refining the clinicopathologic features and molecular and genomic profiling of PTCLs have translated into adjustments and changes introduced in both proposals which are largely overlapping, overall reflecting similar conceptual shifts, with slight differences.

**Fig. 1:**
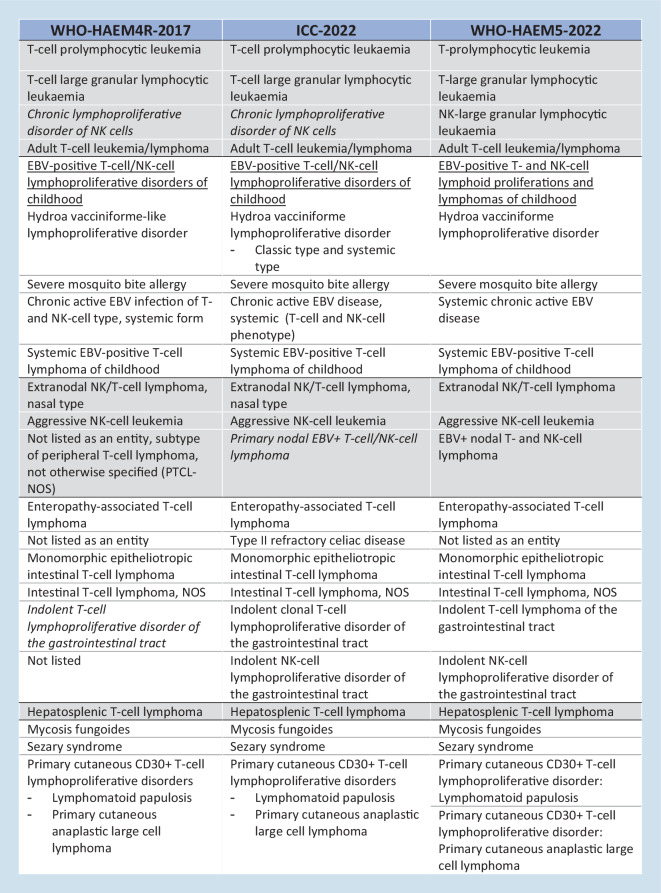
Classification of mature T and NK cell neoplasms in the ICC [5] and WHO-HAEM5 [1] proposals (2022) with reference to the WHO-HAEM4R classification (2017). The entities are listed according to the order in which they appear in the ICC-2022. Colors denote groups of entities. Italics indicate the entities provisional in the WHO-HAEM4R and ICC-2022

**Fig. 1:**
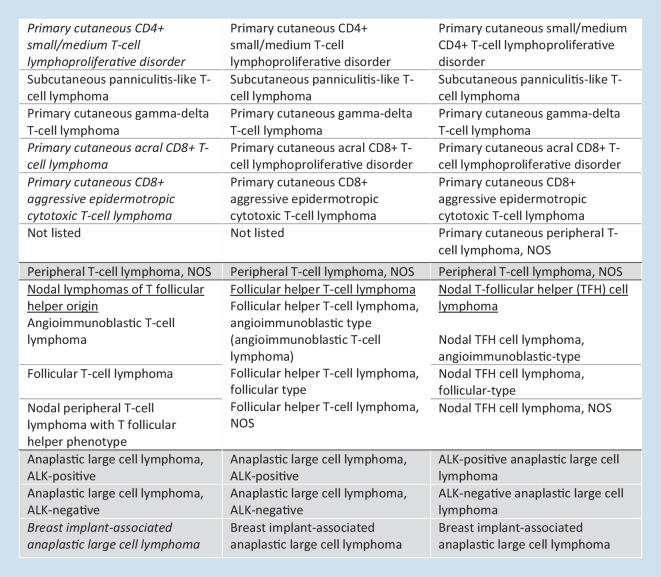
(Continued)

## EBV-associated T cell and NK cell neoplasms

In the group of Epstein–Barr virus (EBV)-driven lymphoproliferative disorders of childhood [21], hydroa vacciniforme lymphoproliferative disorder (LPD) replaces what was previously designated hydroa vacciniforme-like LPD, because essentially all such lesions are associated with EBV infection. The ICC further recognizes two variants: a classic indolent form (limited to the skin) and a systemic aggressive form of the disease, more common in non-Caucasians. Chronic active EBV disease now replaces chronic active EBV infection to denote a pathologic disease condition, in line with the notion that pathogenic mutations indicating a neoplastic process are detected in a subset of patients.

The terminology, definition, and diagnostic criteria of extranodal NK/T cell lymphoma (ENKTCL) nasal type are unchanged in the ICC. Since this lymphoma is known to occur at various extranodal sites besides the nasal area which is involved in typical cases, “nasal type” was dropped in the WHO-HAEM5.

Cases of primary nodal EBV-positive T cell or NK cell lymphoma, formerly considered as a subtype of PTCL, not otherwise specified (NOS), are now categorized as a separate entity, namely primary nodal EBV+ T cell/NK cell lymphoma, provisional in the ICC, or nodal EBV+ T and NK cell lymphoma in the WHO-HAEM5 [21]. This rare disease, most prevalent in East Asia, involves lymph nodes and is frequently disseminated but lacks nasal involvement and tends to occur in elderly adults, in association with HIV infection or immunodeficient conditions [14]. Pathological features distinct from ENKTCL include a monomorphic large cell morphology, less frequent necrosis, negativity for CD56, positivity for CD8, and more frequent derivation from T cells than from NK cells [14, 19]. The tumor is characterized by loss of 14q11.2, upregulation of immune pathways, low genomic instability and recurrent mutations involving the epigenetic modifiers, such as *TET2* and *DNMT3A*, and JAK-STAT pathway genes [27].

## Follicular helper T cell lymphoma

**Fig. 2:**
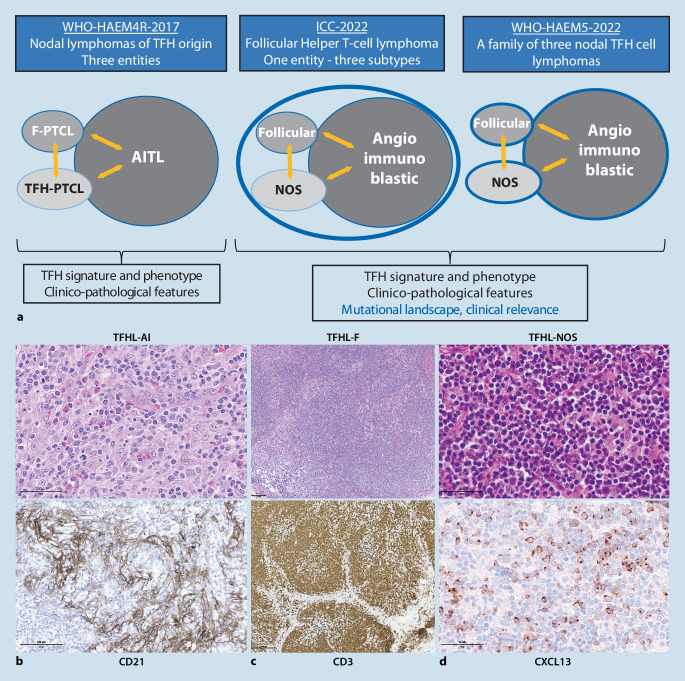
Follicular helper T cell lymphoma. **a** Evolution of the classification of T cell lymphomas of follicular helper T cell (TFH cell) derivation in the successive classifications. **b** Follicular helper T cell lymphoma of the angioimmunoblastic type (TFHL-AI) comprising a polymorphous cellular infiltrate with prominent vessels and expansion of CD21+ follicular dendritic cells. **c** Follicular helper T cell lymphoma of the follicular type (TFHL‑F) is exemplified here by a case showing a follicular lymphoma-like appearance, comprising nodules of CD3+ T cells also positive for several TFH markers (not shown). **d** Follicular helper T cell lymphoma, not otherwise specified (TFHL-NOS), consists of a diffuse lymphoproliferation of atypical CD4+ cells expressing two or more TFH markers, shown here is CXCL13 expression (**b**–**d** HE and immunoperoxidase)

In 2017, the developing concept that follicular helper derivation represents a unifying feature of a large group of nodal CD4+ T cell lymphomas was reflected by the creation of an umbrella term “nodal T cell lymphoma of T follicular helper (TFH) origin” to encompass angioimmunoblastic T cell lymphoma, follicular T cell lymphoma, and nodal PTCL with T follicular helper phenotype. Since then, this notion has been reinforced by additional evidence indicating shared molecular and genetic features [10], and, importantly, clinical data suggest that this grouping might be relevant to treatment decisions, as TFH lymphoma appear more sensitive to epigenetic therapies than non-TFH PTCLs (Fig. 2a; [4]). Therefore, the ICC considers one single disease entity, namely follicular helper T cell lymphoma, comprising three subtypes, angioimmunoblastic, follicular, and NOS (Fig. 2b–d; [11]). This entity by definition excludes primary cutaneous CD4+ T cell lymphoproliferations which also feature a TFH phenotype. The WHO-HAEM5 proposal is more conservative, considering a family of three related entities of nodal T follicular helper cell lymphomas. The TFH immunophenotype is defined by the expression of at least two and ideally three TFH markers out of a panel of at least five markers (CD10, BCL6, PD1, ICOS, CXCL13) that it is now recommended to test for routinely and systematically when a diagnosis of TFH lymphoma is considered or must be excluded [1, 5]. TFH lymphomas frequently carry mutations in *TET2, DNMT3A, RHOA, *and* IDH2*, which are rarely seen in combination in other PTCL entities; hence, mutational testing may be diagnostically useful [8, 12].

## Anaplastic large cell lymphomas

**Fig. 3:**
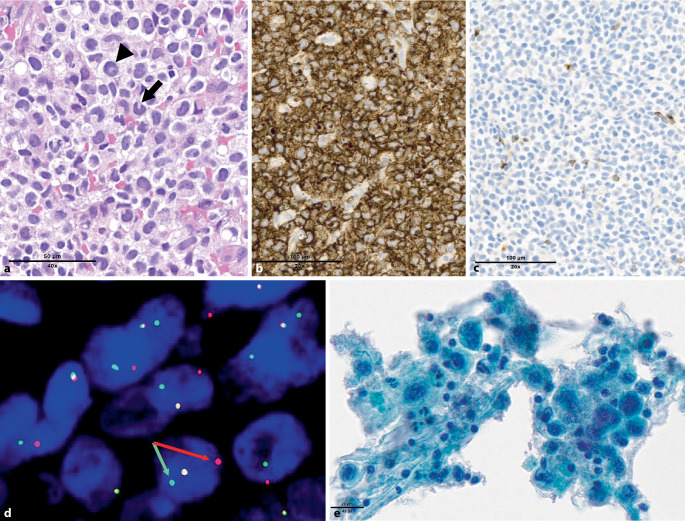
Anaplastic large cell lymphomas. **a**–**d** ALK-negative *DUSP22*-rearranged anaplastic large cell lymphoma, showing a typical morphology including kidney-shaped (*arrow*) and doughnut-shaped (*arrowhead*) tumor cells (**a**), which are strongly and diffusely CD30 positive (**b**), and negative for ALK (not shown) and granzyme B (**c**). Break-apart FISH reveals *DUSP22* gene rearrangement (**d** *red* and *green arrows* point to split signals). **e** Breast implant-associated anaplastic large cell lymphoma diagnosed on a Papanicolaou-stained smear of a periprosthetic effusion, displaying large tumor cells with anaplastic nuclear features, admixed with inflammatory cells

The four entities of anaplastic large cell lymphomas (ALCLs) are identical in both proposals: ALK-positive (ALK+) and ALK-negative (ALK−) ALCL, primary cutaneous ALCL (within the spectrum of CD30-positive cutaneous T cell lymphoproliferative disorders), and breast implant-associated (BIA-)ALCL. Among ALK− ALCLs, those with *DUSP22* rearrangement (25–30% of cases; Fig. 3a–d) differ from those devoid of this alteration, as they usually lack JAK-STAT3 activation and EMA expression, less frequently express cytotoxic molecules, harbor *MSC* mutations in about one third of cases, and have distinctive transcriptomic signature and methylation profiles [17, 18]. The clinical impact of *DUSP22* rearrangement remains controversial: the initially reported markedly superior prognosis of these cases was not confirmed in subsequent studies, while data from more recent cohorts still support an intermediate prognosis of *DUSP22*-rearranged ALK− ALCL, standing between ALK+ ALCL and *DUSP22*-non rearranged ALK− ALCL [23, 24]. Taking into account its biological and prognostic peculiarities, the ICC recognizes *DUSP22*-rearranged ALCL as a genetically defined subtype of the disease and recommends systematic FISH testing for *DUSP22* in ALK− ALCL [8]. Other structural aberrations are recurrent in ALK− ALCL but less common. These include *TP63* rearrangements, associated with an adverse prognosis [23]; as well as fusion genes involving tyrosine kinases such as *JAK2, FRK, ROS1*, and *TYK2*, which may represent potential therapeutic targets [8].

BIA-ALCL (Fig. 3e) is recognized as a definitive entity both in the ICC and WHO-HAEM5. While histopathologically it largely overlaps with systemic ALK− ALCL, the pathogenetic association of BIA-ALCL with the microenvironment of textured breast implants is unique. At the genetic level, a highly characteristic 20q13.13 loss has been reported in two thirds of cases [7], and mutations in epigenetic modifiers such as *KMT2C, KMT2D*, and *CREBBP* are also frequently detected [16]. Similar to systemic ALCL, activation of the JAK-STAT3 pathway is a constant feature of BIA-ALCL, most commonly through mutations of *STAT3* and/or *JAK1* [16]. In contrast, rearrangements of *ALK, DUSP22, *or *TP63* associated with systemic ALCLs are not observed. The prognosis of BIA-ALCL is generally excellent after surgical removal of the periprosthetic fibrous capsule, but is less favorable in cases of infiltration of the adjacent breast parenchyma [15].

## Primary intestinal T and NK cell lymphomas and lymphoproliferative disorders

The three main aggressive types of primary intestinal T cell lymphomas (enteropathy-associated T cell lymphoma [EATL], monomorphic epitheliotropic intestinal T cell lymphoma (MEITL), and intestinal T cell lymphoma, NOS) are unchanged [9]. EATL occurs in populations with a higher prevalence of HLA haplotypes predisposing to celiac disease, as a complication of celiac disease and refractory celiac disease, or de novo in individuals with no history of malabsorption. The tumors may be multiple and present as ulcers or, less commonly, masses, comprise a polymorphous infiltrate with admixed inflammation, and often pleomorphic to anaplastic lymphoma cells. The typical immunophenotype is CD3+ CD4− CD8− CD30+/− TCR-silent EBV-negative, with expression of cytotoxic molecules. MEITL presents as a tumor mass and spans a morphologic spectrum. While typical cases are monomorphic with little necrosis, other tumors exhibit pleomorphic cytology and/or other atypical features like necrosis, brisk mitotic activity, and angiocentricity [26]. In MEITL, the neoplastic cells are CD3+ CD4− CD8+ CD56+ TCR-positive (gamma-delta more commonly than alpha-beta) EBV-negative. Genomic features may be helpful in differentiating between EATL and MEITL: alterations in the JAK/STAT pathway genes target primarily *STAT3* and *JAK1* in EATL, and *STAT5B* and *JAK3* in MEITL. Deleterious alterations of the *SETD2* gene, translating into reduced H3K36 trimethylation, are almost constant and rather specific to MEITL [22]. Type II refractory celiac disease has been added to the list of entities in the ICC, as this represents an “in situ” neoplastic condition precursor to EATL, and recent works have shown that it often already harbors driving mutations in *JAK1* and/or *STAT3 *similar to those present in EATL [6]*.*

**Fig. 4:**
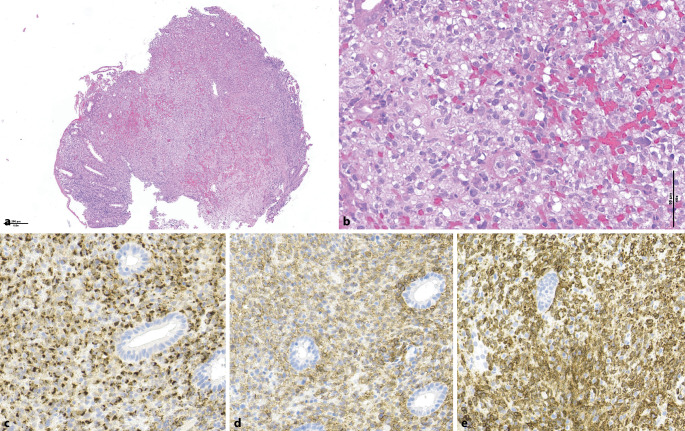
Indolent NK cell lymphoproliferative disorder of the gastrointestinal tract. This colonic biopsy shows a diffuse mucosal infiltrate of atypical lymphoid cells with clear cytoplasm, partially obliterating the crypts (**a** and **b**). The cells are positive for TIA‑1 (**c**), CD56 (**d**), and CD3 (**e**), and were negative for EBV (not shown)

The formerly provisional “indolent T cell LPD of the gastrointestinal tract” is confirmed in the ICC with the addition of “clonal” to emphasize its neoplastic nature. Indeed a variety of somatic genetic alterations have been found in these cases, including a recurrent *JAK2::STAT3* fusion in a subset of CD4+ cases [25]. In WHO-HAEM5, the name has been modified to “indolent T cell lymphoma,” given the fact that transformation into a high-grade PTCL has been described in some patients. Both proposals have created a new category to classify the indolent gastrointestinal LPD of NK cells (Fig. 4), which also carry a variety of genetic mutations, including a recurrent *JAK3 *small in-frame deletion [28]. These T and NK LPDs of the gastrointestinal tract are in general restricted to the mucosa and represent a diagnostic challenge and should not be confused, on the one hand with inflammatory conditions, on the other hand with aggressive lymphomas, since their course is usually indolent despite possible relapses, multifocality, and chronicity, and they do not respond to chemotherapy.

## Peripheral T cell lymphomas, not otherwise specified

**Fig. 5:**
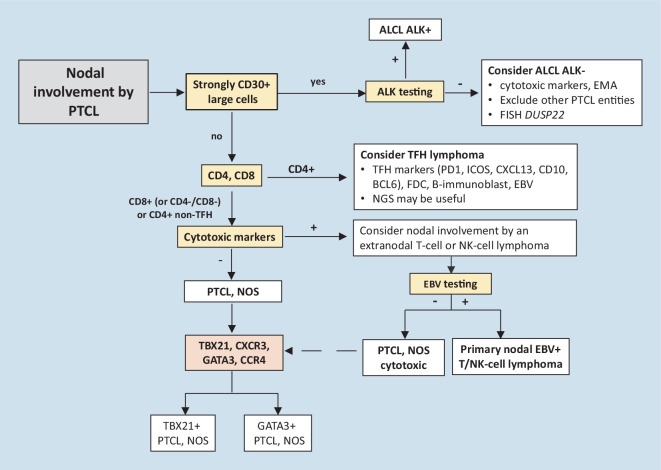
Algorithm for the diagnosis of nodal peripheral T cell lymphomas (PTCLs). (Adapted from [5])

The group of PTCLs, not otherwise specified (NOS), remains a diagnosis of exclusion (Fig. 5). Cases with a TFH immunophenotype must be excluded, since lymphomas with no morphologic specification but showing a TFH immunophenotype, defined by the expression of two or ideally three TFH markers, are classified as TFH lymphoma, NOS. Moreover, caution must be applied in this scenario to exclude primary cutaneous T cell lymphomas or human T-lymphotropic virus type 1 (HTLV-1)-associated adult T leukemia/lymphoma, as these entities, which are often CD4+, may show expression of TFH markers [20].

Two biological subtypes of PTCL, NOS, namely PTCL-TBX21 and PTCL-GATA3, have been identified by gene expression profiling, and are characterized by overexpression of transcription factors TBX21 or GATA3 and corresponding target genes, with different prognoses and distinct oncogenic pathways ([12, 13]; Table 1). An immunohistochemical algorithm using four markers applied sequentially (TBX21, CXCR3, GATA3, and CCR4) can provide surrogate information on the molecular subtypes [3], and a digital nanostring-based assay has recently been published [2]. However, it is acknowledged that there is currently too little evidence to recommend molecular subtyping of PTCL, NOS, in routine clinical use [3]. PTCL-GATA3 demonstrates high genomic complexity characterized by biallelic deletion/mutation of *TP53, CDKN2A/B*, or *RB1, *and carries a worse prognosis compared to PTCL-TBX21, which shows low genomic complexity and few recurrent specific genetic changes.

**Table 1:** Comparison of PTCL-GATA3 and PTCL-TBX21 subtypes of peripheral T cell lymphomas, not otherwise specified (PTCL, NOS) [3, 12]

	PTCL-GATA3 30–40%	PTCL-TBX21 50–60%
Gene expression signature	Th1 like MYC overexpression High proliferation PI3K activation	Th2 like Subset cytotoxic Enrichment of NF-kappa B pathway
Clinical	Poorer outcome	Better outcome Cytotoxic phenotype associated with poorer outcome
Morphology and phenotype	Less inflammatory background GATA3+ and/or CCR4+ (> 50%)	Inflammatory background TBX21+ and/or CXCR3+ (> 20%)
Genomics and gene expression	Higher genomic complexity Genomic aberrations include deletions of 17p (*TP53*), 9p (*CDKN2A*), and 10p (*PTEN*)	Fewer genomic aberrations, targeting cytotoxic effector genes Frequent mutations in epigenetic modulators (e.g., *TET2, DNMT3A*)

## Conclusion

In conclusion, the updated classifications of T and NK cell neoplasms confirm the diversity and complexity of these disorders. Nevertheless, the accumulating knowledge of their biology is translated into more meaningful categories, and an increasing importance of molecular testing for precision diagnosis and tailored therapy.

## Declarations

For this article no studies with human participants or animals were performed by any of the authors.

The supplement containing this article is not sponsored by industry.
